# Radiobiological Assessment of Targeted Radionuclide Therapy with [^177^Lu]Lu-PSMA-I&T in 2D vs. 3D Cell Culture Models

**DOI:** 10.3390/ijms242317015

**Published:** 2023-11-30

**Authors:** Julia Raitanen, Bernadette Barta, Hermann Fuchs, Marcus Hacker, Theresa Balber, Dietmar Georg, Markus Mitterhauser

**Affiliations:** 1 Ludwig Boltzmann Institute Applied Diagnostics, 1090 Vienna, Austria; julia.raitanen@meduniwien.ac.at (J.R.);; 2 Department of Biomedical Imaging and Image-Guided Therapy, Division of Nuclear Medicine, Medical University of Vienna, 1090 Vienna, Austria; 3 Vienna Doctoral School of Chemistry (DoSChem), University of Vienna, 1090 Vienna, Austria; 4 Institute of Inorganic Chemistry, Faculty of Chemistry, University of Vienna, 1090 Vienna, Austria; 5 Department of Radiation Oncology, Division of Medical Radiation Physics, Medical University of Vienna, 1090 Vienna, Austria; 6 Joint Applied Medicinal Radiochemistry Facility, Medical University of Vienna, University of Vienna, 1090 Vienna, Austria

**Keywords:** radiation oncology, radiobiology, multicellular tumor spheroids (3D), targeted radionuclide therapy, [^177^Lu]Lu-PSMA, prostate cancer (PC-3, LNCaP)

## Abstract

In vitro therapeutic efficacy studies are commonly conducted in cell monolayers. However, three-dimensional (3D) tumor spheroids are known to better represent in vivo tumors. This study used [^177^Lu]Lu-PSMA-I&T, an already clinically applied radiopharmaceutical for targeted radionuclide therapy against metastatic castrate-resistant prostate cancer, to demonstrate the differences in the radiobiological response between 2D and 3D cell culture models of the prostate cancer cell lines PC-3 (PSMA negative) and LNCaP (PSMA positive). After assessing the target expression in both models via Western Blot, cell viability, reproductive ability, and growth inhibition were assessed. To investigate the geometric effects on dosimetry for the 2D vs. 3D models, Monte Carlo simulations were performed. Our results showed that PSMA expression in LNCaP spheroids was highly preserved, and target specificity was shown in both models. In monolayers of LNCaP, no short-term (48 h after treatment), but only long-term (14 days after treatment) radiobiological effects were evident, showing decreased viability and reproductive ability with the increasing activity. Further, LNCaP spheroid growth was inhibited with the increasing activity. Overall, treatment efficacy was higher in LNCaP spheroids compared to monolayers, which can be explained by the difference in the resulting dose, among others.

## 1. Introduction

Cancer is the second leading cause of death, after cardiovascular diseases [1]. According to the statistics of the World Health Organization (WHO), the number of new cancer cases in 2020 accumulated to over 19 million and is estimated to rise up to 30.2 million by 2040 [2,3].

Radiotherapy plays a fundamental role in cancer therapy [4]. Currently, around 50% of all cancer patients require radiotherapy, either during primary management or for symptom control in a palliative setting [5]. While external beam radiotherapy (EBRT) is highly effective in primary cancers and solitary metastasis, it is not suited for treating micro metastases. In this context, molecular radionuclide therapy (MRT) is the treatment of choice [6]. The National Institutes of Health (NIH) have predicted that by 2035, radiopharmaceuticals will be used in approximately 60% of all radiation therapy procedures [7].

A current MRT treatment option of metastatic castrate-resistant prostate cancer (mCRPC) is systemic therapy with [^177^Lu]Lu-PSMA-I&T. Due to the high expression of the prostate-specific membrane antigen (PSMA) in this type of tumor and Lu-177 being an ideal radionuclide for the treatment of micro-metastases (mean range of 670 μm and 0.1–2.2 MeV energies of beta particles), [^177^Lu]Lu-PSMA-targeted radionuclide therapy is a highly effective therapy for most mCRPC patients. However, clinical studies show that around 30% of prostate cancer patients do not respond to this form of treatment [8,9].

To further improve MRT, biological research for new or improved vectors and a better understanding of radiation effects is needed [10]. Findings from radiobiology can not only support optimization, but also improve predictions on efficacy and adverse effects [11]. Moreover, radiobiological data are essential for better dose–effect modeling to overcome the common problem of suboptimal therapy delivery [12].

To date, in vitro therapeutic efficacy studies are mostly performed using a two-dimensional (2D) cell culture. Although simple, fast, and well-established, this model fails to properly mimic in vivo tumoral behavior. To overcome this limitation, the first three-dimensional (3D) cell culture model was introduced in 1943 [13]. Multicellular tumor spheroids were found to better recapitulate in vivo tumors in terms of their structure and growth behavior. Their structure is characterized by an inner necrotic core, an adjacent quiescent zone, and a proliferating outer layer, establishing molecular gradients [14,15,16,17]. The phenomenon of hypoxia and, thereof, resulting necrosis was previously shown to be apparent in many in vivo tumors [18].

In previous studies, the cell killing efficacy of Ra-223 [19], Ac-225 [20], Pb-212 [21], and Cu-64 [22] with different carriers was investigated in spheroids, showing significant detrimental effects.

In this study, we showcased an evaluation of the short- and long-term radiobiological effects of the clinically established [^177^Lu]Lu-PSMA-I&T therapy using 2D and 3D cell culture models. Herein, we present several methods and describe the observed differences between the models.

## 2. Results

### 2.1. Verification of PSMA Expression via Western Blot

**Figure 1:**
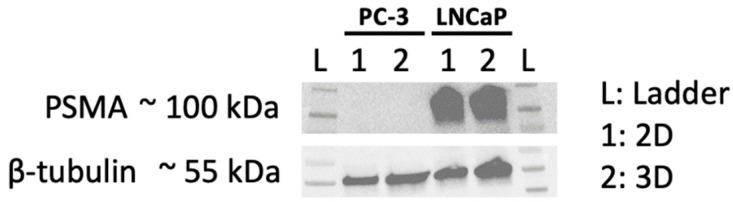
Western Blot of 2D and 3D samples of PC-3 and LNCaP cell lines; target = PSMA, loading control = β-tubulin.

To verify conserved target expression upon growth in spheroids, cell lysates from monolayers and spheroids were prepared and blotted. As depicted in Figure 1, the PSMA-negative cell line, PC-3, showed no band in 2D and 3D, while high PSMA expression was demonstrated in both models of the LNCaP cell line.

### 2.2. Trypan Blue Exclusion Assay: Short-Term Cell Viability and Replicability in Monolayers

To investigate short-term cell viability and proliferation in monolayers, a dye exclusion assay was performed directly after treatment and 48 h after treatment over the range from 0 to 2 MBq for 3 h on LNCaP and PC-3 cells. No significant change in cell viability or cell number with increasing activity was observed: neither directly (0 h after treatment), nor 48 h after treatment with up to 2 MBq [^177^Lu]Lu-PSMA-I&T for 3 h (Supplementary Figure S1).

### 2.3. Evaluation of Reproductive Potential in Monolayers by Standard Clonogenic Assay

**Figure 2:**
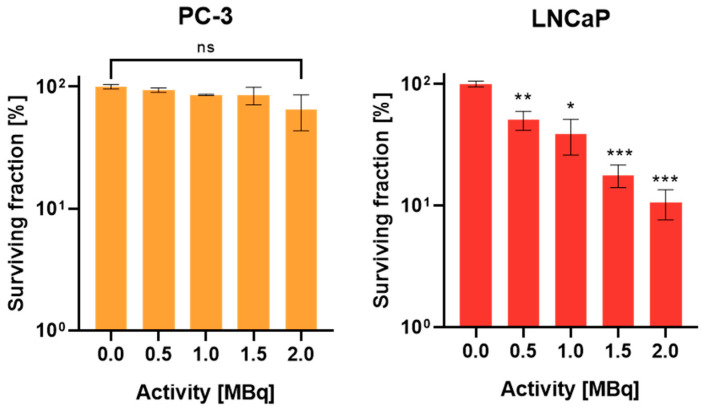
Survival of PC-3 (left) and LNCaP (right) cells; plotted as mean ± standard deviation from three to four independent experiments (n = 3–4) performed in triplicates. Statistical difference compared to control (ns = not significant, * = *p* < 0.005, ** = *p* < 0.001, *** = *p* < 0.0001).

To further evaluate the reproductive potential of cells cultivated in monolayers, clonogenic assays were performed. Figure 2 depicts a significant decrease in the surviving fraction with increasing activity in PSMA+ (PSMA positive) LNCaP cells. In contrast, PSMA- (PSMA-negative) PC-3 cells showed no significant change in the surviving fraction over the range from 0 to 2 MBq [^177^Lu]Lu-PSMA-I&T treatment for 3 h.

### 2.4. Growth Analysis of Spheroids

**Figure 3:**
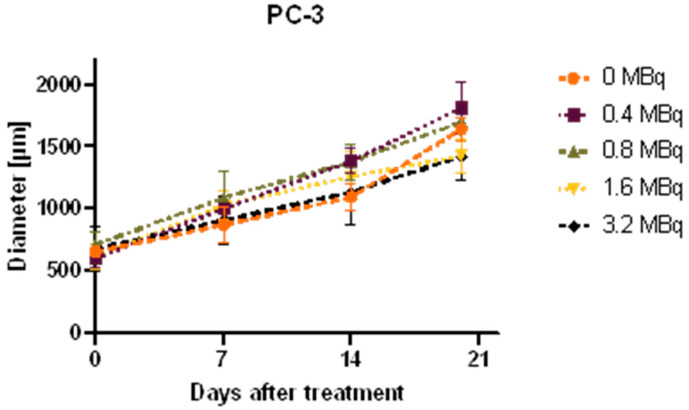

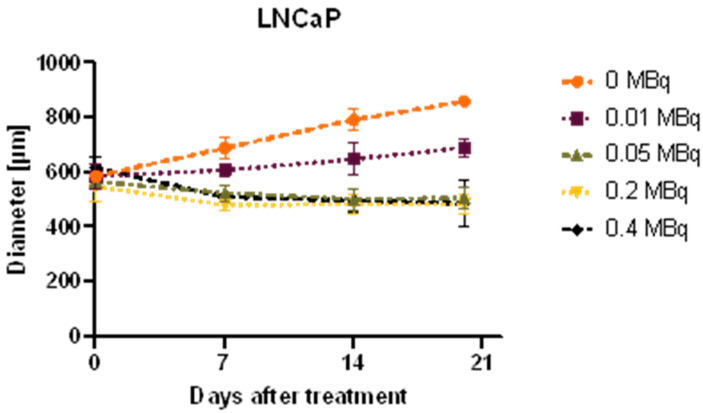
Growth observation of PC-3 (top) and LNCaP (bottom) spheroids after treatment. Diameter of spheroids; average over three diameter measurements per spheroid and time point are plotted as mean ± standard deviation from three independent experiments (n = 3).

While PC-3 spheroids showed no significant difference in size compared to the control up to 3.2 MBq, significant growth inhibition was evident in LNCaP spheroids already at the lowest applied dose of 0.01 MBq. Treatment with 0.05 MBq [^177^Lu]Lu-PSMA-I&T showed a high treatment effect and was not significantly different to 0.2 or 0.4 MBq. Spheroid diameters 0, 7, 14, and 20 days after treatment are depicted in Figure 3. The spheroid shape and growth rate were cell line dependent. Time lapse images are given in the supplement (Supplementary Figure S2).

### 2.5. Long-Term Cell Viability Determination Using Cell Titer Glo^®^: 2D vs. 3D

**Figure 4:**
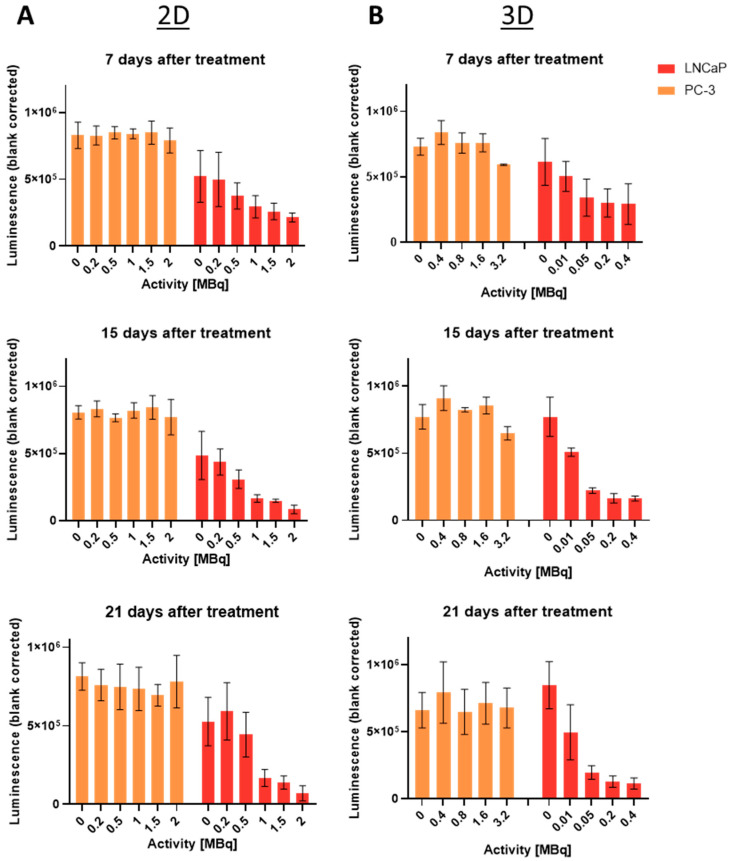
Cell viability assessment in 2D (**A**) and 3D (**B**) models of PC-3 and LNCaP cells on days 7, 15, and 21 after treatment, using Cell Titer Glo^®^ 3D reagent. Plotted as mean ± standard deviation from three to four independent experiments (n = 3–4) in duplicates. Luminescence signal corresponds with viability. Note the differences in applied activity between 2D and 3D.

By means of the Cell Titer Glo^®^ assay, ATP was measured as a surrogate for viability, detected by a luminescent readout. Figure 4 displays the cell viability comparison of 2D and 3D models of PC-3 and LNCaP cells. Neither monolayers nor spheroids of PC-3 showed a significant decrease in viability after treatment compared to the control, evident on day 7, 14, and 21 after treatment. For LNCaP, a significant decrease with increasing activity was detected. However, the viability in the LNCaP spheroids decreased already at much lower doses, compared to the monolayers. Further, the difference to the control increases over time. Please note the differences in the applied activity.

On day 7 after treatment with 0.2 MBq [^177^Lu]Lu-PSMA-I&T for 3 h, a 2.4-fold difference between 2D and 3D was observed. This difference further increased over time, reaching a factor of 4.5 on day 21 after treatment, as shown in Table 1.

### 2.6. Dosimetry Assessment Using Monte Carlo Simulations

**Figure 5:**
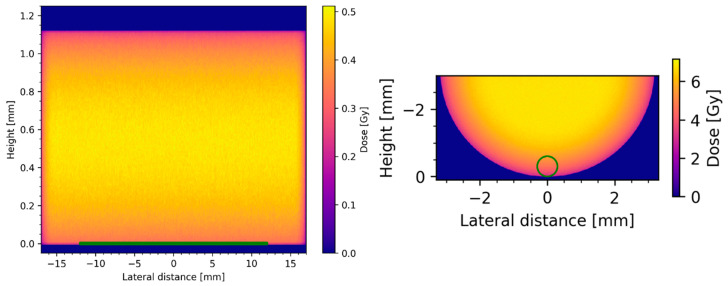
Visualization of dose distribution in 2D (**left**) and 3D (**right**) models. Green line/circle depicts monolayer/spheroid scoring region.

To assess the influence of a geometrical effect on the resulting absorbed dose (in Gy) at the different models, Monte Carlo (MC) simulations were performed. Simulations were only used to assess the deposited dose on a macroscopic level. As shown in Figure 5, the cell monolayer was modeled using a cylinder of a 12 mm diameter and 0.01 mm height, and for the 3D spheroids, a sphere with a diameter of 0.6 mm was used. The surrounding medium was modeled by water and assuming evenly distributed activity for the treatment of 3 h.

Depending on the geometry of wells and models, considerable gradients were observed in the absorbed dose. Using an activity of 1 MBq and 3 h of treatment duration, the scoring region (depicted in green) in the 2D setting received 0.25 Gy, while the scoring region in the 3D setting received 4.54 Gy in the same conditions. A conversion table from the applied activity to the resulting absorbed dose is given in the supplement (Supplementary Table S1).

## 3. Discussion

In vitro radiobiological assessments of new therapeutics are currently mostly performed in 2D since it is affordable and already well-established. However, the lack of resemblance to in vivo tumors can lead to wrong estimations of efficacy. Multicellular tumor spheroids can enhance the predictive power and minimize the error by being a better mimic, ultimately reducing time and costs during drug development by the earlier detection of ineffective agents [23]. Thus, 3D models are credited to be fundamental tools in pharmaceutical development and biomedical research in the future [23,24].

This in vitro study investigated the differences in cell viability and proliferation between the 2D and 3D models of the two prostate cancer cell lines, PC-3 and LNCaP, after treatment with the already clinically used radioligand therapy agent, [^177^Lu]Lu-PSMA-I&T. Thus, this study elucidates the differences between monolayers and spheroids in respect to radioligand therapy and provides the methodology for radiobiological studies of targeted radioligand therapy in spheroids.

After confirming preserved high PSMA expression in LNCaP spheroids and monolayers, which is the basis for the application of this targeted radionuclide therapy, we investigated cell viability and replicability in monolayers using a Trypan Blue Exclusion assay. No decrease in viability and cell number up to 48 h was observed, suggesting no effects of senescence or apoptosis in the first two days after treatment. However, as expected, a decrease in the surviving fraction with increasing activity was evident for the target expressing LNCaP cells after 14 days (clonogenic assay), but not in PSMA negative PC-3 cells, demonstrating the specificity of the therapeutic agent.

In 3D models, growth monitoring showed no treatment effect in the PSMA-lacking PC-3 cells, but significant growth inhibition over 20 days in PSMA+ LNCaP, confirming target specificity also in the 3D model. Interestingly, a significant treatment effect was evident already at the lowest activity of 0.01 MBq. Increasing the treatment from 0.05 MBq to 0.4 MBq showed no increase in growth inhibition. We assume that already with 0.05 MBq, most of the cells in the spheroid are either killed or senescent. In addition, we hypothesize that the higher dose of 0.4 MBq leads to different repair mechanisms, which let the cells survive in a similar extent as after treatment with 0.05 MBq.

The comparison of 2D and 3D in one assay was performed by means of a Cell Titer Glo^®^ viability assay. When analyzing long-term cell viability after treatment with [^177^Lu]Lu-PSMA-I&T, LNCaP spheroids were more effectively treated than the respective monolayers, apparent by the strong decrease in viability already at lower applied activities than in 2D. After treatment with 0.2 MBq [^177^Lu]Lu-PSMA-I&T for 3 h and observation for 7 days afterwards, cell viability was 2.4-fold higher in 2D, compared to 3D. Over time, this effect increased, reaching a 4.5-fold difference on day 21 after treatment.

In order to evaluate the extent of the geometric effects, resulting from the large beta particle range of Lu-177, Monte Carlo simulations were performed. Our results showed that the cell monolayer received only about 0.25 Gy while the spheroids received 4.54 Gy (corresponding to 1 MBq for 3 h), explaining the lower viability in 3D versus 2D. As shown before [25], Monte Carlo simulations could in principle be used to go beyond the macroscopic dose distribution employed in this manuscript and provide insights into the micro- and nanodosimetric scale. However, such models are still under investigation and no clear recommendations have been published so far. When comparing these findings to our previously published results [26], where we irradiated 2D and 3D models of the same cell lines with X-ray, the calculated absorbed doses for the applied activities fit well into the respective clonogenic survival curves previously published, validating the simulation.

A possible explanation for the increase in the sensitivity of spheroids to radionuclide therapy over time could be a retention of the compound in the 3D construct, leading to an extended treatment duration with a concomitant elevated bystander effect. However, it remains to be investigated whether the extent of this effect is similar to in vivo tumors.

## 4. Materials and Methods

### 4.1. Chemicals and Reagents

All chemicals and reagents were purchased from Sigma-Aldrich (St. Louis, MO, USA) unless stated otherwise.

### 4.2. Cell Culture

The cell lines of PC-3 (PSMA-negative: PSMA-) and LNCaP (PSMA-positive: PSMA+) were obtained from the American Type Culture Collection (ATCC) and cultivated in Roswell Park Memorial Institute (RPMI) 1640 medium. Medium was supplemented with 10% fetal bovine serum, 2 mM Glutamine, and 1% penicillin (10,000 U/mL)/streptomycin (10,000 µg/mL). All cell culture reagents were obtained from Gibco Life Technologies Ltd. (Paisley, UK). Culturing took place at 37 °C with 5% CO_2_ and cells were routinely tested for mycoplasma contamination using the MycoAlert™ Mycoplasma Detection Kit (Lonza, Basel, Switzerland), according to the manufacturer’s instructions [27].

### 4.3. Generation of Spheroids

Spheroids were generated as previously described [26], using the liquid overlay technique. In short, 2000 cells/well (in 100 µL medium) were seeded into U-bottom 96-well plates, which were precoated with a thin layer of 1.5% low gelling agarose. LNCaP were cultured scaffold-free. For PC-3 spheroids, 3% Geltrex^TM^ (LDEV-Free Reduced Growth Factor Basement Membrane Matrix, Gibco) was added to the cell suspension. Plates were centrifuged at 161× *g* for 10 min at 4 °C before further incubation at 37 °C and 5% CO_2_.

### 4.4. Western Blot

Whole cell lysates were prepared to assess the amount of prostate-specific membrane antigen (PSMA) in 2D and 3D cultures. Then, 2D and 3D lysate preparation as well as Western Blot were performed as described previously [26].

Membranes were incubated with the following primary antibodies: anti-PSMA, (D4S1F; Cell Signaling Technology Europe B.V., Leiden, The Netherlands; 1:1000 in 5% DMP in TBS-T) or anti-β-Tubulin (Cell Signaling Technology; 1:1000 in 5% BSA in TBS-T) overnight at 4 °C. After incubation with the respective horseradish peroxidase (HRP)-conjugated secondary antibodies for 1 h at room temperature, detection of chemiluminescence (Pierce^TM^ ECL Western Blotting Substrate Kit; Thermo Fisher Scientific, Waltham, MA, USA) was performed using a ChemiDoc Imaging System (Bio-Rad Laboratories Inc., Berkeley, CA, USA), according to the manufacturer [28].

### 4.5. Treatment Procedure

[^177^Lu]Lu-PSMA-I&T (10 MBq/nmol) was provided by the routine production at the General Hospital of Vienna. Monolayers and spheroids were treated for 3 h in either 1 mL (2D) or 100 µL (3D) medium with activities up to 3.2 MBq (total activity per well). Medium was discarded and renewed after treatment. Spheroids were additionally washed with 100 µL DPBS before adding 100 µL of fresh medium. Medium was renewed once per week until the last experiment, 3 weeks after treatment.

### 4.6. Microscopic Growth Analysis

An Olympus IMT-2 Inverted Microscope with an XC50 Color Camera (Olympus, Tokyo, Japan) and cellSens Entry (Version 2.3, Olympus) software were used to monitor spheroid growth and obtain diameter measurements on days 0, 7, 14, and 20 after treatment. One representative spheroid per cell line and activity was chosen for each experiment and a mean value of three diameter measurements per spheroid was calculated. Data were then plotted in Prism Version 8 (GraphPad, San Diego, CA, USA).

### 4.7. Clonogenic Assay

By use of standard clonogenic assays, the reproductive ability after radiation treatment was determined [29]. For this, 250 PC-3 or 1000 LNCaP cells were seeded in 6-well plates in 2 mL medium per well and incubated overnight. On the following day, plates were treated with 0.5, 1, 1.5, or 2 MBq [^177^Lu]Lu-PSMA-I&T per well. PC-3 cells were further cultured for 7 days and LNCaP for 14 days, with medium being changed once per week. After washing with PBS, cells were fixed with methanol for 30 min at 4 °C. This was followed by another washing step and final staining with crystal violet (Sigma-Aldrich; 1% in water) for 3 min. Excess stain was removed by submerging the plates in tap water and stained colonies were counted by eye.

Plating efficiency was determined by dividing the average number of colonies in the control plate by the number of total seeded cells. To obtain the surviving fraction (SF), the plating efficiency was multiplied by the number of colonies after treatment.

### 4.8. Trypan Blue Exclusion Assay

For evaluation after 0 h, 500,000 cells of both cell lines, and for evaluation after 48 h, 100,000 PC-3 or 250,000 LNCaP were seeded in 6-well plates. After overnight incubation, cells were treated with 0.5, 1, 1.5, and 2 MBq per well (in 1 mL medium) for 3 h. 0 and 48 h after treatment; supernatants, wash fractions, and harvested cells were combined and centrifuged for 4 min at 161× *g* at room temperature. Supernatants were discarded, and cell pellets were resuspended in 0.5 mL medium. For determination of cell viability and cell number, single cell suspensions were mixed with Trypan blue (1:1; Gibco) and assessed with a LUNA^TM^ automated cell counter (Logos Biosystems, Anyang, Republic of Korea).

### 4.9. Viability Assessment Using Cell Titer Glo^®^ 3D

The assay was performed according to the manufacturer’s protocol [30]. For 2D assessment, 4000 PC-3 or 40,000 LNCaP cells were seeded in 6-well plates, incubated overnight and treated with up to 3.2 MBq of [^177^Lu]Lu-PSMA-I&T (day 0). On day 6, supernatant and detached cells of control and each treatment condition were collected and counted, hereby supernatant was not discarded, but combined with the detached cells to also retrieve dead/detached cells. Further, the liquid volume to seed a total of 5000 cells was calculated from the control. This calculated volume was seeded for all treatment conditions (in total 100 μL/well) in white-walled 96-well plates and incubated overnight. To assess viability on day 15, 4000 PC-3 or 40,000 LNCaP cells were again seeded in 6-well plates and incubated until day 14. The same procedure was applied for viability assessment on day 21. This procedure needed to be done in order to prevent overgrowth in the control plates, but still providing enough cells for viability analysis in the condition with the highest activity treatment. On days 7, 15, and 21, plates and reagent were first equilibrated to room temperature for 30 min before addition of 100 µL reagent per well. This was followed by vigorous shaking of the plate for 5 min using the Synergy HTX multimode reader (Biotek, Winooski, VT, USA). After another incubation at room temperature for 25 min, luminescence was measured in the same reader. For 3D viability assessment, spheroids were transferred to white-walled 96-well plates on days 7, 15, and 21. Reagent addition and measurement was performed as described for monolayers.

### 4.10. Dosimetry

Monte Carlo (MC) simulations were performed using GATE v9.3 alongside GEANT4 v11.1. GATE is an open-source general-purpose MC tool-kit, developed by the OpenGATE collaboration since 2001 [31,32]. Both 2D as well as 3D wells were modeled in GATE based on the respective manufacturer’s cell vessel specifications, using 1 mm-thick polystyrene walls. In the simulation, medium was modeled by water and the treatment was applied as specified above. Activity was evenly distributed within the water volumes. To improve simulation efficiency, the Lu-177 decay was modeled solely using electron sources with the corresponding energies (78.6% 497 keV, 9.1% 384 keV, and 12.2% 176 keV). Scoring regions approximating the corresponding biological samples were designed as follows: for the 2D cell layer, a cylinder of 12 mm diameter and 0.01 mm height; for the 3D spheroid, a sphere with a diameter of 0.6 mm. Voxel size was set to 0.01 mm in all directions for the 3D well, while for the 2D well, a voxel size of 0.1 × 0.1 × 0.01 mm was used to improve the simulation performance while remaining at maximum resolution. Uptake and retention into the 2D or 3D cell structures could not be considered during simulations. Absorbed dose (in Gy) in the scoring regions was averaged over the whole scoring volume. Simulations were performed to achieve a statistical variance below 1.5%. MC parameters are listed in Table 2.

### 4.11. Statistical Analysis

Data analysis was performed in Prism software version 8 (GraphPad, San Diego, CA, USA). For data comparison, multiple unpaired *t*-tests were performed and *p*-values < 0.01 were considered statistically significant.

## 5. Conclusions

We investigated the differences in radiobiological response when testing radionuclide therapy in monolayers and spheroids. Our results showed that the PSMA-targeted radionuclide therapy is specific for LNCaP in both models and more effective in 3D compared to 2D. MC simulations showed that geometric effects lead to higher doses for the 3D model compared to the monolayers.

## Figures and Tables

**Table 1:** Differences in cell viability in LNCaP between 2D and 3D after treatment with 0.2 MBq [^177^Lu]Lu-PSMA-I&T for 3 h and observation on Day 7, 14, and 21.

Days after Treatment	X-Fold Difference (2D/3D)
7	2.4
14	2.9
21	4.5

**Table 2:** MC parameters used for dosimetric simulations.

Parameter	Value
Physics list	QGSP_BIC_HP_EMZ
Ionization potential of water	78 eV
Production and stopping cuts in scoring region	0.01 mm
Maximum step size in scoring region	0.01 mm

## Data Availability

Data supporting reported results can be provided upon request.

## Supplementary Materials

The following supporting information can be downloaded at: https://www.mdpi.com/article/10.3390/ijms242317015/s1.
