# Supplementary material for: Radiobiological Assessment of Targeted Radionuclide Therapy with [177Lu]Lu-PSMA-I&T in 2D vs. 3D Cell Culture Models
Source: Int J Mol Sci. 2023 Nov 30;24(23):17015. doi: 10.3390/ijms242317015 (PMC10706939; doi:10.3390/ijms242317015)
Supplement: Supplementary file 1 [file ijms-24-17015-s001.zip › ijms-2708544-supplementary.pdf]

Supplementary Information

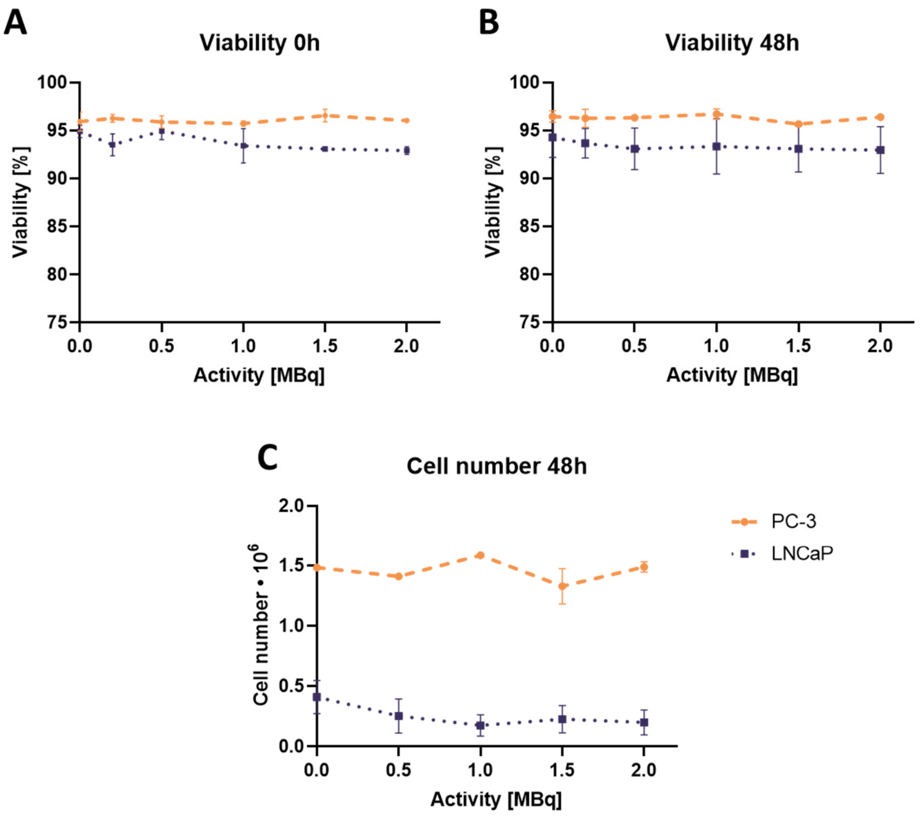

Figure S1. Trypan Blue Exclusion Assay: evaluation of cell viability and proliferation in monolayers. Cell viability immediately (0 h; (**A**)) and 48 h (**B**) after treatment. Cell count 48 h (**C**) after treatment. Graphs are plotted as mean ± standard deviation from two independent experiments (n = 2) performed in duplicates.

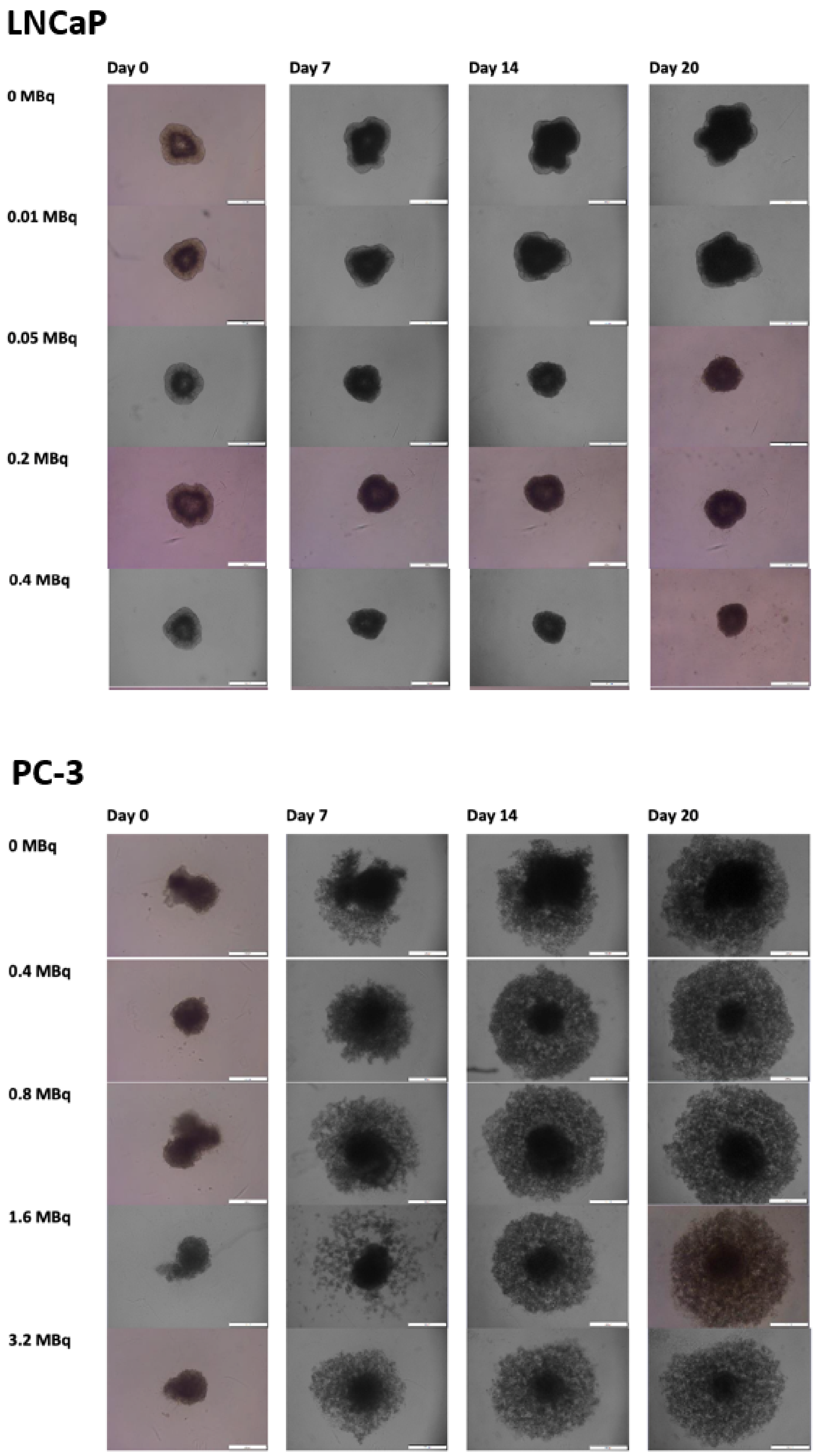

Figure S2. Time lapse growth observation of LNCaP and PC-3 spheroids treated with up to 3.2 MBq on day 0; white scale indicates 500 µm.

Table S1. Conversion table from applied activity to resulting dose.

Applied Activity [MBq]	Resulting Dose 2D [Gy]	Resulting Dose 3D [Gy]
0.01	0.002	0.05
0.05	0.01	0.23
0.2	0.05	0.91
0.4	0.10	1.82
0.5	0.13	2.27
0.8	0.20	3.63
1.0	0.25	4.54
1.5	0.38	6.81
1.6	0.40	7.26
2.0	0.50	9.08
3.2	0.80	14.53
